# The association of socio-demographic characteristics of university students and the levels of their digital entrepreneurial competences

**DOI:** 10.1016/j.heliyon.2023.e20897

**Published:** 2023-10-12

**Authors:** Vladimir Simovic, Ivana Domazet, Milica Bugarcic, Mirna Safi, Hamsa Sarhan, Rupali Bhagat, Aleksandra Bradic Martinovic

**Affiliations:** a Australian University, Kuwait / Institute of Economic Sciences, Serbia; b Institute of Economic Sciences, Serbia; c Belgrade Banking Academy, Serbia; d Australian University, Kuwait

**Keywords:** Digital entrepreneurial competences, EmDigital, University students competence assessment, Identification of opportunities

## Abstract

The aim of this article is to provide empirical evidence on the level of digital entrepreneurial competences (DEC) that higher education students acquire during their higher education and to investigate the relationship between their socio-demographic characteristics and the level of DEC. The first competence area (Identification of opportunities) of the DEC EmDigital competence framework was assessed together with its three sub-competence areas (Search for and analysis of information, Creativity and innovation, and Prospecting), and the relationship between the level of DEC and various socio-demographic characteristics of higher education students was investigated using logistic regression. The results show a relatively low DEC level of the participating students for the first EmDigital competence area. Location, field of study, level of study and employment status were found to be statistically significant for the acquisition of DEC, while the variables age and gender had little impact. The results of this study are a step towards the development of an DEC online assessment tool (using the Digital Competence Wheel as a role model) that universities and other stakeholders could use to assess the level of DEC their students acquire during university education. This research is the first study to assess the acquisition of DEC during university studies and the impact of different socio-demographic characteristics of students on their level of DEC.

## 1 Introduction

The dynamic nature of today's society requires a new approach to educating our future professionals that takes into account global economic development and the demands of a turbulent labour market [1]. New models that enable students to acquire knowledge, skills and attitudes suitable for a real work environment relevant to them should replace the old model based on knowledge transfer and memorisation. In addition to new educational models, special attention should be paid to the new types of skills and competences relevant to today's labour market. Zhao et al. [2] suggest that higher education institutions should be encouraged to focus on developing students' and teachers' digital competences, developing relevant learning strategies and using appropriate tools to improve the quality of education. Information and communication technology (ICT) has transformed a wide range of entrepreneurial processes and outcomes, e.g. by facilitating the operations of start-ups, mediating new businesses and entrepreneurial activities, and enabling new digital business models [3,4]. As a result, new competences are emerging to harmonise the digital transformation of entrepreneurship [5].

Academic studies and business practise, as well as public policy, aim to promote digital entrepreneurship for its positive impact on job creation and economic growth, and to understand the circumstances and causes that foster it [6]. Various societal actors believe that building both entrepreneurial [7] and digital competences [8] is crucial to properly address digital innovation. In addition to physical resources, digital entrepreneurs need a variety of digital competences to make the best use of technologies for the most impactful digital innovation [9]. Digital entrepreneurs need skills and competences that support the identification and exploitation of opportunities based on technical knowledge [10]. Consequently, digital entrepreneurial competences (DEC), which are a specific combination of general digital and entrepreneurial competences [11], are gaining importance and becoming the focus of the research community.

Melnikova et al. [12] define DEC as “the total ability of the entrepreneur to perform a job role successfully using a range of ICT means”. DEC is the combination of entrepreneurial competences [13,14] and ICT competences [15,16] that shape a digital entrepreneur's start-up choices and post-entry strategic decisions [17]. Competences include knowledge and behavioural skills associated with entrepreneurs' goals, business activities and tasks [18,19]. Chou et al. [20] identify the following hard skills as components of DEC: (1) entrepreneurial competences, (2) marketing competences, (3) business and economic competences, (4) financial competences, (5) accounting affairs competences, (6) management competences, (7) globalisation competences, (8) business law competences, (9) enterprise resource planning competences and (10) information technology competences. According to the DEC framework developed by Prendes-Espinosa et al. [21] called EmDigital, DEC consists of the following four competence areas: Identification of opportunities, Action planning, Initiative and collaboration and Management and safety. The competence areas are divided into 15 sub-competence areas as follows: Search for and analysis of information, Creativity and innovation, Prospecting, Success orientation, Leadership, Planning and management of digital identity, Initiative, Communication and collaboration, Creation of digital value, Responsibility and commitment, Learning from experience, Problem solving, Planning and organization, Techno-ethical approach, and Motivation and perseverance.

The growing interest in DEC is a direct result of the expansion of digital entrepreneurship [22]. Although the topic of digital entrepreneurship is relevant and timely, there is little literature on the relationship between education and digital entrepreneurship and the importance of education in building DEC. Some authors [23] analyse the methodological aspects of maturity levels and DEC development programme and propose an algorithm for selecting an educational method based on achieving the desired outcomes from the perspective of the maturity level of university students DEC. Ngoasong [16] explores how context as an antecedent of DEC influences digital entrepreneurship in a resource-scarce environment. A study by Thanachawengsakul [24] identifies three levels of DEC: the use of digital technology for collaboration, work and digital accessibility and awareness. Kruger & Steyn [25] analyse the conceptual model of DEC, which is required for the use of Industry 4.0 technologies. Most of the recent literature on university students DEC focuses on examining the relationship between DEC and digital entrepreneurial intention (DEI). Alferaih [26] explores the impact of digital entrepreneurship education as a key prerequisite for acquiring DEC on DEI of university students. Singh & Dwivedi [27] investigate the relationship between DEC, DEI and entrepreneurial motivation (EM) in a sample of university students in India. Their findings suggest that DEC is a critical determinant of the development of DEI and eventually EM. These findings are consistent with a large number of studies that have shown that “traditional” entrepreneurship education programmes aimed at developing the entrepreneurial competences (EC) of university students have an impact on their entrepreneurial intention (EI) [[28], [29], [30], [31], [32], [33], [34]]. Given the positive relationship between EC and EI, empirical studies have been conducted to assess the level of EC of university students [35] and to relate the level of EC with various socio-demographic characteristics of university students [36]. The main purpose of these studies was to investigate the success of university entrepreneurship education in creating EC as a crucial determinant for the development of students' EI. No evidence was found in the academic literature that research has been conducted to measure the level of DEC for any target group. Some attempts were made by Tekin et al. [37] to develop a scale to measure the entrepreneurial competences of university students in the digital age, using Kailer\s Entrepreneurship Competence Model. However, no evidence for its use and no empirical results were found.

Universities around the world are developing new and adapting existing business and IT programmes to provide their students with the highest possible level DEC, so that they are better prepared for today's labour market [38]. The problem lies in the fact that no one knows or has ever tried to measure the DEC level that university students acquire during their education. The lack of an adequate methodology and assessment tools is the main reason for this. Simovic & Domazet [11] propose the development of the DEC framework and an appropriate methodology to measure the level of DEC that students acquire during their formal university education. The ultimate goal is to develop an online DEC assessment tool using the best examples of competence assessment tools, such as the Digital Competence Wheel [39].

In their structured literature review, Secundo et al. [40] propose the creation of entrepreneurial competences in digital “university-based” entrepreneurial ecosystems as one of the four main research areas for future research on digital entrepreneurship. The available data on the association of students DEC and DEI [26,27] and the obvious analogy with “traditional” entrepreneurship and the association between EC and EI call for research that assesses the level of DEC (similar to Ref. [35]) and relates the level of DEC to various socio-demographic characteristics of university students (similar to Ref. [36]). This research is necessary to show that digital entrepreneurship education at the university level is successful in creating DEC as a crucial determinant for building students DEI. In the same vein, Alferaih [26] suggests that future research could use demographic variables such as age, gender, income, etc. as moderating variables to see how they influence students' intentions to start a business after they graduate and reach a certain level DEC. In their integrative literature review on academic digital entrepreneurship, Garcez et al. [41] identify several research gaps in the field of digital entrepreneurship, including measuring the level of DEC at the time of leaving the university programme. The aim is to evaluate the effectiveness of the methods used during university education.

This paper fills the gap in the literature (the lack of studies measuring the level of DEC) by providing empirical evidence of the actual level of DEC of university students, using the DEC competence framework EmDigital [21], which is a basis for the creation of the DEC assessment tool. This study provides the first empirical evidence of the level of university students DEC and its relationship with various socio-demographic characteristics of students. More specifically, the research presented here addresses the first competence area of EmDigital, Identification of opportunities. It examines the relationship between the three sub-competences in this competence area and the socio-demographic characteristics of university students.

Given the above challenges, three research questions were posed:

RQ1 What is the DEC level of university students in the first competence area of EmDigital, Identification of opportunities?

RQ2 Is the level of DEC in the first competence area of EmDigital related to the socio-demographic characteristics of university students?

RQ3 What is the association between the three sub-competences in the first competence area of EmDigital, Identification of opportunities, and the different socio-demographic characteristics of university students?

## 2 Literature review

### 2.1 Digital entrepreneurial competences

A competence, as described by various authors, is a process that enables people to solve problems creatively, perform activities, formulate questions, search for and analyse relevant information, and analyse, interpret and reflect while applying their knowledge to real-world needs [[42], [43], [44], [45]]. In terms of student learning and competences, modern education has undergone a paradigm shift. For example, the European Higher Education Area (EHEA) places the concept of competence at the centre of the learning process and students at the centre of the educational mode. These changes have led to a new educational paradigm that places a higher value on competences than on content, because the inclusion of competences means more than just the transmission of information; it also means a commitment to improving the inseparable relationship between university courses and the skills and knowledge needed in the labour sector. Therefore, academics should ensure that they include the skill profiles required and specified for career success and performance in their competency-based learning environment [46].

Innovation and digitalisation have transformed the work environment worldwide, leading to a change in the work competences and skills required in the market. It has been noted [47] that sometimes there is a skills and competency gap because the workforce lacks the required competences and skills. Higher education institutions should not ignore this fact and work to provide training in digital entrepreneurship to young potential entrepreneurs [48], as this is a necessary competence not only for their education but also for their lives [21]. Every university or academic institution today needs to train competitive experts with creative entrepreneurial thinking who are able to develop innovative projects for today's digitalised economy [23]. Graduates are expected to cope with changes in the local and global economy, and digital and entrepreneurial competences will help them become more employable than students who do not have these competences [49]. Developing entrepreneurial competences and skills will help the young workforce to be educated and determine their labour market [50].

DEC are the knowledge, ability and potential to create and develop new business concepts solely through the use of digital means and the use of the virtual world [51]. According to Ngoasong [16], DEC is the knowledge and skills required to find and acquire new information, identify and exploit entrepreneurial opportunities and innovate. Fayolle & Benoit [52] suggest that DEC can be acquired through formal education, context-specific training and some prior experience. In line with this argument, Kraus et al. [53] state that the key competences for digital entrepreneurship are acquired in the education system. Digital entrepreneurship education is one of the knowledge transfer processes in creating entrepreneurs [54]. Digital entrepreneurship education is the integration of digital knowledge and entrepreneurial skills. Digital education can be used to develop soft skills in each learner to help them improve their employability in line with the current Industrial Revolution 4.0 [55]. Unlike entrepreneurship education, which is one of the fastest growing fields in the world [56,57] research on digital entrepreneurship education is obscure. It is mainly concerned with the role of MOOCs in digital entrepreneurship education [58,59], the impact of COVID -19 on the reorganisation of digital entrepreneurship teaching [60] and game-based learning in digital entrepreneurship education [61].

DEC represent a specific combination of general digital and entrepreneurial competences [11] and a relatively new area of interest in the academic community due to the rapid growth of digital entrepreneurship in the world, especially during and after the Covid 19 pandemic [62]. It is well known that extreme situations such as the pandemic create new entrepreneurial opportunities and change risk perceptions [63].

### 2.2 Competence frameworks and assessment tools

The most important prerequisite for the development of competence assessment tools is the existence of relevant competence frameworks. Understanding DEC requires a thorough understanding of general digital and entrepreneurial competences, and assessing DEC requires the use of best practises for general digital and entrepreneurial competences in the form of competence frameworks and related assessment tools [11].

General digital competences are nowadays a crucial factor for any kind of business success. Digital competences are recognised by the European Commission [64] as one of eight key competences and have been defined as the confident and critical use of Information Society Technology (IST) for work, leisure and communication. Basic ICT skills such as using computers to retrieve, assess, store, produce, present and exchange information and to communicate and participate in collaborative networks via the Internet form the basis for this. Digital competences are the focus of various competence frameworks, such as the Digital Competence Framework for citizens - DigComp [65], which recognises 21 digital competences divided into five competence areas. Another framework is the e-Competence Framework, which provides a reference of 40 digital competences at five proficiency levels that describe the competences, skills and knowledge requirements of ICT professionals in ICT business [66].

There are many tools/tests to assess digital skills such as the Digital Competence Wheel [39], Digital Skills Accelerator (five organisations from Poland, Belgium, Spain, UK and Ireland), Ikanos (Spain) and SmartiveMAP (Italy). Kuziminska et al. [67] studied the digital competences of students and teachers in Ukraine using the Digital Competence Wheel and found that the competence levels for professional use of IT are much higher among students than among teachers. Lopez-Meneses et al. [1] studied the digital competences of students from one Italian and two Spanish universities in three areas of DigComp 2.1: information and data literacy, communication and collaboration, and digital content creation.

According to Barot [68], entrepreneurship “practice begins with action and creation of the new organisation.” Entrepreneurship can also be seen as “the ability to turn ideas into action through creativity, innovation and risk-taking by planning projects to achieve certain objectives” [64]. Entrepreneurship is considered an elementary competence for the development of every human being, as it represents a personal commitment to the improvement of our environment [21].

In order to improve the entrepreneurial capacity of European citizens and organisations, the European Commission's Joint Research Centre (JRC), on behalf of the Directorate-General for Employment, Social Affairs and Inclusion (DG EMPL), created the Entrepreneurship Competence Framework, also known as EntreComp [69]. EntreComp is the framework that highlights the importance of entrepreneurship and illustrates how people can use entrepreneurial competence to address various economic, social and cultural challenges. EntreComp treats entrepreneurship as a competence for life where individuals seek opportunities in every situation, from school to seeking innovative solutions in the workplace. In its basic form, EntreComp encompasses three competence areas: Ideas and Opportunities, Resources and Into action. Each area includes five competences, resulting in a total of 15 competences, all of which have the same value [70].

Examples of entrepreneurial skills assessment tests/tools are the OctoSkills app (previously ASTEE, Denmark), ESP (Entrepreneurial Skills Pass, JA Europe) and HEInnovate (European Commission, DG Education and Culture and the OECD LEED Forum).

In early 2021, a group of Spanish researchers [21] published an article in which they developed a DEC framework called EmDigital. The framework is based on the digital competence framework DigComp and the entrepreneurial competence framework EntreComp. The EmDigital framework is divided into four competence areas and 15 specific competences. In developing EmDigital, Prendes-Espinosa et al. [21] used a qualitative methodology based on an interpretive approach. Their work to create the EmDigital framework included document analysis, focus groups and expert judgment.

Prendes-Espinosa et al. [21] found that there is a gap between the level of DEC that students are expected to acquire in higher education institutions and the actual level of DEC. There is no evidence in the academic literature that research has ever been conducted to measure the level of DEC that students acquire during their higher education and to investigate the relationship between the level of DEC and students' socio-demographic characteristics.

## 3 Empirical method

### 3.1 Design

The quantitative approach was adopted for this study. Data were collected from a sample of university students in Kuwait and Serbia using a questionnaire. Descriptive and inferential statistics (logistic regression) were used to assess the level of DEC of university students and to investigate the relationship between different socio-demographic characteristics of university students and their level of DEC. Logistic regression was selected as an efficient method to analyse the impact of a group of socio-demographic characteristics of university students on a binary outcome (specific DEC level) by quantifying the unique contribution of each socio-demographic characteristic.

### 3.2 Instrument

The EmDigital framework proposed by Prendes-Espinosa et al. [21] was used to develop the instrument to assess the level of university students DEC. The research focused on the first competence area of EmDigital, called Identification of opportunities. This competence area consists of three specific sub-competences and six indicators, as presented in Table 1 in the Appendix.

**Table 1:** Descriptive statistics.

Question	Answer	Full Sample	Country		Level of study		
			Serbia	Kuwait	Bachelor	Diploma	Master
EQ1	FALSE	70.5	71.8	68.8	63.5	83.0	63.6
	TRUE	29.5	28.2	31.3	36.5	17.0	36.4
EQ2	FALSE	59.1	48.2	73.4	52.9	75.5	27.3
	TRUE	40.9	51.8	26.6	47.1	24.5	72.7
EQ3	FALSE	71.1	60.0	85.9	60.0	88.7	72.7
	TRUE	28.9	40.0	14.1	40.0	11.3	27.3
EQ4	FALSE	50.3	55.3	43.8	51.8	45.3	63.6
	TRUE	49.7	44.7	56.3	48.2	54.7	36.4
EQ5	FALSE	55.0	51.8	59.4	52.9	54.7	72.7
	TRUE	45.0	48.2	40.6	47.1	45.3	27.3
EQ6	FALSE	85.9	82.4	90.6	78.8	94.3	100.0
	TRUE	14.1	16.5	9.4	20.0	5.7	0.0
EQ7	FALSE	70.5	60.0	84.4	57.6	90.6	72.7
	TRUE	29.5	40.0	15.6	42.4	9.4	27.3
EQ8	FALSE	51.7	41.2	65.6	44.7	66.0	36.4
	TRUE	48.3	58.8	34.4	55.3	34.0	63.6
EQ9	FALSE	70.5	72.9	67.2	74.1	66.0	63.6
	TRUE	29.5	27.1	32.8	25.9	34.0	36.4
EQ10	FALSE	49.7	29.4	76.6	43.5	66.0	18.2
	TRUE	50.3	70.6	23.4	56.5	34.0	81.8
EQ11	FALSE	91.9	89.4	95.3	89.4	96.2	90.9
	TRUE	8.1	10.6	4.7	10.6	3.8	9.1
EQ12	FALSE	60.4	62.4	57.8	55.3	64.2	81.8
	TRUE	39.6	37.6	42.2	44.7	35.8	18.2
Mean	FALSE	65.5	60.4	72.4	60.4	74.2	63.6
	TRUE	34.5	39.5	27.6	39.5	25.8	36.4
St. deviation	FALSE	13.7	17.0	15.1	13.9	16.4	24.8
	TRUE	13.7	17.2	15.1	14.1	16.4	24.8

Question	Answer	Field of study			Employment status		Gender	
		Finance	IT	Business economy	Employed	Unemployed	Male	Female
EQ1	FALSE	72.2	72.4	69.6	73.7	69.4	74.2	67.8
	TRUE	27.8	27.6	30.4	26.3	30.6	25.8	32.2
EQ2	FALSE	27.8	55.2	65.7	39.5	65.8	67.7	52.9
	TRUE	72.2	44.8	34.3	60.5	34.2	32.3	47.1
EQ3	FALSE	66.7	44.8	79.4	65.8	73.0	75.8	67.8
	TRUE	33.3	55.2	20.6	34.2	27.0	24.2	32.2
EQ4	FALSE	27.8	65.5	50.0	55.3	48.6	54.8	47.1
	TRUE	72.2	34.5	50.0	44.7	51.4	45.2	52.9
EQ5	FALSE	50.0	51.7	56.9	50.0	56.8	45.2	62.1
	TRUE	50.0	48.3	43.1	50.0	43.2	54.8	37.9
EQ6	FALSE	83.3	75.9	89.2	84.2	86.5	85.5	86.2
	TRUE	16.7	20.7	10.8	15.8	12.6	14.5	12.6
EQ7	FALSE	66.7	58.6	74.5	57.9	74.8	74.2	67.8
	TRUE	33.3	41.4	25.5	42.1	25.2	25.8	32.2
EQ8	FALSE	50.0	41.4	54.9	31.6	58.6	59.7	46.0
	TRUE	50.0	58.6	45.1	68.4	41.4	40.3	54.0
EQ9	FALSE	61.1	79.3	69.6	65.8	72.1	69.4	71.3
	TRUE	38.9	20.7	30.4	34.2	27.9	30.6	28.7
EQ10	FALSE	27.8	27.6	59.8	23.7	58.6	56.5	44.8
	TRUE	72.2	72.4	40.2	76.3	41.4	43.5	55.2
EQ11	FALSE	88.9	93.1	92.2	89.5	92.8	90.3	93.1
	TRUE	11.1	6.9	7.8	10.5	7.2	9.7	6.9
EQ12	FALSE	55.6	69.0	58.8	52.6	63.1	56.5	63.2
	TRUE	44.4	31.0	41.2	47.4	36.9	43.5	36.8
Mean	FALSE	56.5	61.2	68.4	57.5	68.3	67.5	64.2
	TRUE	43.5	38.5	31.6	42.5	31.6	32.5	35.7
St. deviation	FALSE	20.9	18.3	13.4	19.9	12.6	13.4	15.2
	TRUE	20.9	18.6	13.4	19.9	12.7	13.4	15.4

The approach proposed by Kluzer and Priego [71] was used in the development of the instrument (questionnaire). These authors developed an assessment tool based on DigComp to measure the digital competences of the adult population. It was based on the use of knowledge and ability questions (KA-Q), where individuals are confronted with real problems in a variety of real-life situations and have to indicate what they would do in a given situation, what would happen in reality, etc. This approach measures factual knowledge (knowing that …) and procedural knowledge (knowing how to perform certain tasks) or both.

The questionnaire was first prepared in English (for Kuwaiti students) and then translated into Serbian (for Serbian students), with minor changes to adapt it to the Serbian context. The introductory part of the questionnaire collected basic socio-demographic data about the respondents (country, age, gender, level of study and field of study). In the second part of the questionnaire, data was collected on the three sub-competence areas of the Identification of opportunities (Search for and analysis of information, Creativity and innovation, and Prospecting). For each specific indicator (Table 1 in the Appendix) proposed by Prendes-Espinosa et al. [21], we developed a set of 2 questions. Our questionnaire includes 12 questions - 2 questions per specific competence indicator (see Table 2 in the Appendix for a complete list of questions and their codes). Six questions were designed to assess Search for and analysis of information, four questions to assess Creativity and innovation, and two questions to assess Prospecting as sub-competence areas of Identification of opportunities.

**Table 2:** Model parameters for EQ1 and EQ2 (indicator C1.1.).

Parameter		EQ1				EQ2			
		B	Wald	p	Odds ratio	B	Wald	p	Odds ratio
Country	Kuwait	0.423	6.705	0.010	1.526	−0.448	8.764	0.003	0.639
Level of study	Diploma	−1.417	84.455	0.000	0.243	−0.702	25.777	0.000	0.496
	Master	0.383	2.614	0.106	1.466	0.762	9.582	0.002	2.142
Field of study	IT	−0.165	0.531	0.466	1.297	−1.214	31.205	0.000	0.320
	Bus. Econ.	0.260	1.371	0.242	0.848	−1.140	29.095	0.000	0.297
Empl. status	Unemployed	0.335	3.899	0.048	1.399	−0.421	6.983	0.008	0.656
Gender	Female	0.166	1.736	0.188	0.181	0.317	6.939	0.008	1.372
Age		−0.034	6.543	0.011	0.967	0.015	1.751	0.186	1.015
Constant		−0,349	0750	0,387	0706	0,762	4046	0,044	2142
Log-likelihood		−847,238				−891,769			
p		0,000				0000			
Cox & Snell R Square		0,074				0145			
Nagelkerke R Square		0,105				0195			

The following criteria were used to formulate the KA -Q in the second part of the questionnaire:

● Four possible answers for each question
● Four types of answers:
  ○ One correct statement that the respondent should indicate.
  ○ One incorrect statement.
  ○ One distractor, i.e. a false but plausible (not absurd) statement which, if selected, highlights the respondent's inaccurate knowledge
  ○ Do not know the answer, an option introduced to minimise respondent guessing.
● Negative (e.g. “is not something”), ambiguous or vague statements are avoided, as are any grammatical or logical “hooks” that can easily lead to the correct answer. These are the qualitative measures to control method bias (proposed by Podsakoff et al. [72] during data collection.

### 3.3 Data collection and ethical considerations

The survey was conducted simultaneously in Kuwait and Serbia between June 2021 and January 2022. The questionnaire was created using Google Forms and distributed to students in Kuwait during the Summer and Fall semesters and to students in Serbia during the Fall semester of the 2021/22 academic year. The questionnaires were distributed to the students by the teachers in class after explaining the purpose of the study to the participating students.

Various inclusion and exclusion criteria were applied to participants. Only final-year diploma, bachelor and master students of Finance, IT and Business Economy from Kuwait and Serbia were eligible to participate in the study. The reason for excluding students who are not in their final year of study is that the main purpose of the study was to assess the level of DEC of the students at the end of an educational cycle (diploma, bachelor or master). Finance, IT and Business Economy students were included in the study because these students learn digital entrepreneurship (entrepreneurial and ICT skills and competences) during their studies. In Kuwait, only students from universities where English is the primary language of instruction were eligible to participate in the study, while in Serbia, only students from universities where Serbian is the primary language of instruction were eligible to participate. Both female and male students, as well as unemployed and employed students, were eligible to participate in the study.

The study was conducted according to the guidelines of the Declaration of Helsinki and received ethical approval from the Scientific Research Centre of the Australian University in Kuwait (SRC01-05/2021) before it was conducted. The study was anonymous and participating students were informed that they were giving consent to participate in a study by clicking the Continue button in the questionnaire.

### 3.4 Description of the participants

A total of 1551 students attempted to complete the questionnaire. The data was cleaned in the following phase and checked for missing and incomplete responses as well as statistical outliers. The complete responses of 48 respondents were removed as they had not answered all the questions in the questionnaire. Case-wise or list-wise deletion is a common data cleaning practise to avoid data bias in cases where missing values account for more than 50 % of the study variables, and deleting cases does not affect the power of the analysis [73]. Responses were deleted for 11 respondents when it was determined that they had just been straightlining as per Kim et al. [74], and for two respondents when the outliers for the numeric variable age were identified. Once the data had been cleaned, the next step was to process data from 1490 respondents, which formed a final sample size.

**Fig. 1:**
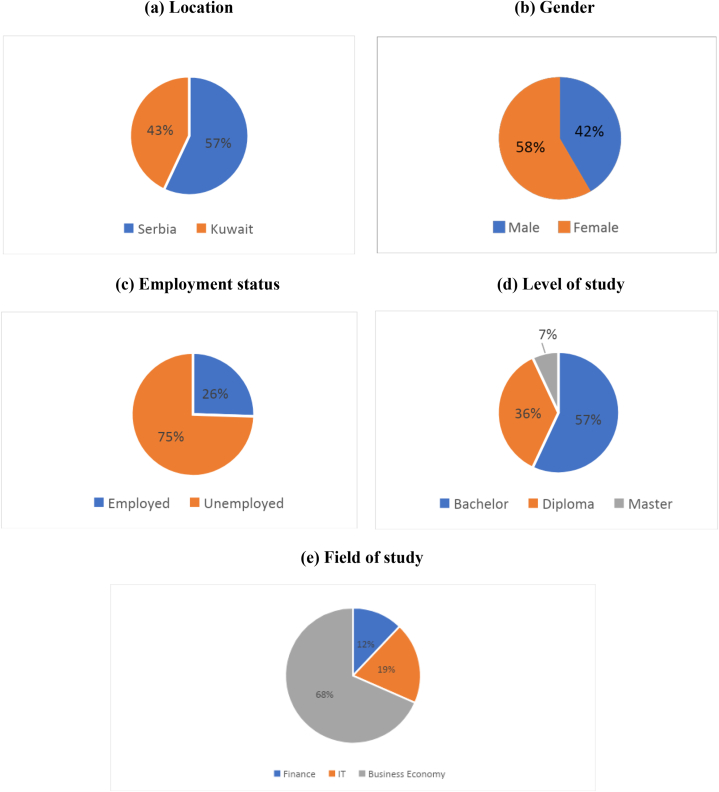
Descriptive statistics.

The sub-sample of students from Serbia was 850, while the sub-sample of students in Kuwait was 640. Male students participated with 42 % of the total sample, while 58 % of the participants were female students. In terms of level of study, Bachelor's students dominated the total sample with 57 %, while Master's students represented 7 % of the total sample. More than two-thirds of the respondents were Business Economy students (68 %). Fig. 1 shows a graphical representation of the sample.

### 3.5 Data analysis

The statistical package SPSS 25.0 was used to analyse the collected data.

The results of the descriptive statistics for all 12 questions (Table 1) show that more than half of the participants knew the answer to questions EQ4 and EQ10, while the lowest number of correct answers was for question EQ11. The fact that some students knew the answers to certain questions and others could not answer certain questions led to the logical inquiry of what factors determine whether students know the correct answer as a measure of their acquired DEC. In this research, it was assumed that the level of acquired DEC depends on certain socio-demographic characteristics of university students, such as country (location), gender, level of study, employment status, field of study and age.

Logistic regression [75] is an efficient and powerful method to analyse the effect of a group of independent variables on a binary outcome by quantifying the unique contribution of each independent variable. If the above socio-demographic characteristics of the participants are marked with X1, X2, …, Xk and the binary variable Y taking the value 0 if the answer is incorrect (all answers were marked as incorrect if respondents selected one of the three answer types in the questionnaire: wrong statement, incorrect but plausible (not absurd) statement or “Do not know the answer” statement) and taking the value 1 if the answer is correct, then the logit model can be used to assess whether or not the student knows the correct answer to a particular question.

(1) $$\ln\ln\left(\frac{\pi }{1-\pi }\right)=\alpha +\beta_{1}X_{1}+\beta_{2}X_{2}+\ldots +\beta_{k}X_{k}$$

This model of logistic regression is used to investigate the impact of the six socio-demographic characteristics of the participants (university students) on the acquired levels of DEC:

$$X_{1},=\text{Country}\;\left(1-\text{Serbia};\;2-\text{Kuwait}\right)\text{,}$$

$$X_{2}=\text{level}\;\text{of}\;\text{studies}\;\left(1-\text{Bachelor};\;2-\text{Diploma};\;3-\text{Master}\right)$$

$$X_{3}=\text{Field}\;\text{of}\;\text{studies}\;\left(1-\text{Finance};\;2-\text{IT};\;3-\text{Business}\;\text{Economy}\right)$$

$$X_{4}=\text{Employment}\;\text{status}\;\left(1-\text{Employed};\;2-\text{Unemployed}\right)$$

$$X_{5}=\text{Gender}\;\left(1-\text{Male};\;2-\text{Female}\right)\text{,}$$

$$X_{6}=\text{Age}$$

The model of logistic regression which can be presented as follows:

(2) $$\ln\ln\left(\frac{\pi }{1-\pi }\right)=\alpha +\beta_{1}X_{1}+\beta_{2}X_{2}+\beta_{3}X_{3}+\beta_{4}X_{4}+\beta_{5}X_{5}+\beta_{6}X_{6}$$

was applied in regression analysis of the acquired DEC of university students in Kuwait and Serbia.

### 3.6 Reliability and validity

Validity refers to the ability of an instrument to measure what it is intended to measure [76], while the reliability refers to the capacity of an instrument to repeatedly measure the intended concept or phenomenon and yield identical results [77].

To achieve validity of the questionnaire and the data collected, a pilot test was conducted with a sample of 150 respondents prior to the main study to ensure that the questions were worded in such a way that they would be understood similarly by respondents from different countries, levels and fields of the study.

To test the internal consistency reliability, the Cronbach's alpha coefficient was calculated. The value of the Cronbach's alpha coefficient is 0.8575, which means that the scale developed in this study is a reliable measurement tool. To check the validity, we conducted a confirmatory factor analysis, which showed that the scale has good goodness-of-fit values. Based on the Kaiser-Meyer-Olkin test for sampling adequacy (0.804) and the p-value together with Bartlett's Test of Sphericity (0.000), we can conclude that a reliable instrument has been developed to measure DEC.

Two pseudo R-squared values (Cox & Snell R-squared and Nagelkerke R-squared) were used to estimate the usefulness of the model. They show what percentage of the variance of the dependent variable (EQ1-EQ12) is explained by each model.

## 4 Results and discussion

The results and discussion section of this paper is structured to provide answers to the research questions posed in Section 1 (Introduction).

### *4.1* DEC level of university students in the first competence area of EmDigital, identification of opportunities

Table 1 presents data on the proportion of correct and incorrect answers for all 12 questions in a full sample of respondents and sub-samples formed according to the socio-demographic variables studied. These data represent the first empirical evidence of the level of DEC (Identification of opportunities) that students acquire during their university education. For a full sample of respondents, the mean values of the proportion of correct/incorrect answers (34.5 %/65.5 %) indicate a relatively low level of acquired DEC (Identification of opportunities) for final-year diploma, bachelor and master students in two countries. The fact that students answered every third question correctly (34.5 % of correct answers) suggests that digital entrepreneurship education and DEC require more attention and a different approach in university education today, at least in the countries that participated in this research. There is no other research on DEC levels of university students to compare these data.

The data shows that students in Serbia perform better compared to students in Kuwait. The level of acquired DEC (identification of opportunities) is higher for bachelor students than for diploma and master students. In terms of field of study, Finance students showed better knowledge than IT and Business Economy students. Employed students performed better than unemployed students, and female students performed slightly better (3.2 %) than male students.

The fact that students in Serbia performed better than their counterparts in Kuwait (39.5 % correct answers in Serbia versus 27.6 % in Kuwait) indicates differences between the two education systems. The fact that university students in Kuwait had lower performance can be linked to the reform failure of the education system in that country over the last two decades [78].

A higher level of DEC (Identification of opportunities) for bachelor's (39.5 %) and master's (36.8 %) students compared to diploma students (25.8 %) can be expected due to a longer education cycle (2 years for a diploma compared to 3–4 years for bachelor's students and five years for master's students). This result is in line with similar research conducted for other competence areas showing that bachelor's degree students have higher competence levels than diploma students [79].

The fact that Finance students scored better (43.5 %) than IT (38.5 %) and Business Economy (31.6 %) students suggests that digital entrepreneurship, and in particular digital entrepreneurship opportunity recognition, is taught more effectively to Finance than to IT and Business Economy students, and/or that their curricula cover the fundamental DEC more effectively or in more detail.

Employed students performed better (42.5 %) than unemployed students (31.6 %), which can be explained by their work experience having a positive impact on their DEC level. This is in line with the findings of Ferreras-Garcia et al. [46], who demonstrated that variable experience has a positive impact on entrepreneurial competences.

Female students performed slightly better (35.7 %) than male students (32.5 %), but the difference is not significant. This observation is in line with similar research by Ferreras-Garcia et al. [80] and Kakkonen [81]. They confirmed that gender has a very little impact on the entrepreneurial competences of female and male students.

### *4.2* Association of DEC levels under the first competence area of EmDigital with socio-demographic characteristics of university students

Data on socio-demographic characteristics of university students in Kuwait and Serbia, collected simultaneously in both countries, were used for parameter estimation of the logistic regression model using the maximum likelihood estimation method.

The empirical results are presented in Table 2, Table 3, Table 4, Table 5, Table 6, Table 7. A separate analysis was conducted for each of the six competence indicators (see Table 1 in the Appendix) under the first competence area (Identification of opportunities) of EmDigital to better understand the association of each DEC indicator with the socio-demographic characteristics of the participating students, as the proportion of correct/incorrect responses for the different indicators suggested a different association of the socio-demographic variables and the corresponding indicators. In addition to the estimated parameter values, the tables also contain data on the p-value (to determine the statistically significant impact on the dependent variable), the Wald coefficient (to determine which variable has the most significant unambiguous impact on the dependent variable), the B coefficient (to calculate the likelihood for the case analysed to belong to a particular category) and the odds ratio (to calculate the likelihood that the dependent variable will change if the independent variable changes). The p-values show that the estimated models are statistically significant (p < 0.0005). The comparison of the estimated models shows that the pseudo R2 value was between 0.030 (question EQ9) and 0.320 (question EQ10).

**Table 3:** Model parameters for EQ3 and EQ4 (indicator C1.2.).

Parameter		EQ3				EQ4			
		B	Wald	p	Odds ratio	B	Wald	p	Odds ratio
Country	Kuwait	−1.000	34.236	0.000	0.368	0.597	17.551	0.000	1.817
Level of study	Diploma	−1.147	46.466	0.000	0.318	0.116	0.821	0.365	1.123
	Master	−0.849	11.822	0.001	0.428	−0.202	0.756	0.385	0.817
Field of study	IT	0.841	0.495	0.482	1.160	−1.540	51.106	0.000	0.250
	Bus. Econ.	0.148	15.577	0.000	2.320	−1.385	44.426	0.000	0.214
Empl. status	Unemployed	0.223	1.725	0.189	1.249	0.277	3.142	0.076	1.319
Gender	Female	0.398	8.543	0.003	1.488	0.257	5.138	0.023	1.292
Age		0.020	2.731	0.098	1.020	0.011	1.066	0.302	1.012
Constant		−1352	11.849	0.001	0,259	0.322	0.796	0.372	1.380
Log-likelihood		−773.949				−981.689			
p		0,000				0000			
Cox & Snell R Square		0,150				0066			
Nagelkerke R Square		0,215				0088

**Table 4:** Model parameters for EQ5 and EQ6 (indicator C1.3.).

Parameter		EQ5				EQ6			
		B	Wald	p	Odds ratio	B	Wald	p	Odds ratio
Country	Kuwait	−0.490	11.526	0.001	0.612	−0.500	5.397	0.020	0.606
Level of study	Diploma	−0.005	0.001	0.969	0.995	−1.308	34.412	0.000	0.270
	Master	−1.174	23.193	0.000	0.309	−20.051	0.000	0.996	1.000
Field of study	IT	−0.268	1.736	0.188	0.962	0.016	0.004	0.952	1.018
	Bus. Econ.	−0.038	0.038	0.845	0.765	0.018	0.004	0.947	1.016
Empl. status	Unemployed	−0.472	9.132	0.003	0.624	−0.061	0.077	0.782	0.941
Gender	Female	−0.778	46.530	0.000	0.459	−0.292	3.009	0.444	0.747
Age		−0.005	0.192	0.661	0.995	0.013	0.586	0.083	1.013
Constant		1.086	9.087	0.003	2.963	−1.324	6.394	0.011	0.266
Log-likelihood		−980.806				−531.923			
p		0.000				0.000			
Cox & Snell R Square		0,058				0071			
Nagelkerke R Square		0,077				0129

**Table 5:** Model parameters for EQ7 and EQ8 (indicator 2.1.).

Parameter		EQ7				EQ8			
		B	Wald	p	Odds ratio	B	Wald	p	Odds ratio
Country	Kuwait	−1.334	60.723	0.000	0.264	−0.970	42.710	0.000	0.379
Level of study	Diploma	−1.792	100.689	0.000	0.167	−0.513	15.053	0.000	0.599
	Master	−1.371	29.838	0.000	0.254	−0.468	3.923	0.048	0.626
Field of study	IT	0.316	8.103	0.003	2.842	0.551	7.187	0.007	2.393
	Bus. Econ.	1.044	2.738	0.147	1.372	0.872	3.411	0.041	1.735
Empl. status	Unemployed	−0.701	1.583	0.175	0.496	−0.873	29.865	0.000	0.417
Gender	Female	0.045	0.108	0.743	1.046	0.448	14.714	0.000	1.565
Age		−0.042	10.339	0.001	0.959	−0.002	0.048	0.827	0.998
Constant		0.916	5.090	0.024	2.499	0.306	0.708	0.400	1.358
Log-likelihood		−762.940				−939.816			
p		0.000				0,000			
Cox & Snell R Square		0.173				0,116			
Nagelkerke R Square		0.246				0,155

**Table 6:** Model parameters for EQ9 and EQ10 (indicator 2.2.).

Parameter		EQ9				EQ10			
		B	Wald	p	Odds ratio	B	Wald	p	Odds ratio
Country	Kuwait	0.456	8.220	0.004	1.578	−1.669	114.792	0.000	0.188
Level of study	Diploma	0.325	5.622	0.018	1.384	−0.119	0.689	0.406	0.888
	Master	0.517	4.838	0.028	1.677	0.323	1.288	0.256	1.382
Field of study	IT	−0.877	15.772	0.000	0.416	0.201	0.780	0.377	1.222
	Bus. Econ.	−0.754	13.165	0.000	0.470	−0.120	0.307	0.580	0.887
Empl. status	Unemployed	−0.323	3.882	0.049	0.724	−0.944	29.083	0.000	0.389
Gender	Female	−0.159	1.683	0.195	0.853	0.308	5.983	0.014	1.361
Age		0.011	0.975	0.323	1.011	0.009	0.556	0.456	1.009
Constant		−0.501	1.834	0.176	0.606	1.095	7.469	0.006	2.988
Log-likelihood		−881.662				−827.967			
p		0.000				0.000			
Cox & Snell R Square		0.030				0.240			
Nagelkerke R Square		0.042				0.320

**Table 7:** Model parameters for EQ11 and EQ12 (indicator 3.1.).

Parameter		EQ11				EQ12			
		B	Wald	p	Odds ratio	B	Wald	p	Odds ratio
Country	Kuwait	−0.867	10.478	0.001	0.420	0.078	0.285	0.593	1.082
Level of study	Diploma	−1.273	20.465	0.000	0.280	−0.544	17.194	0.000	0.581
	Master	−0.318	0.686	0.408	0.728	−1.769	38.906	0.000	0.170
Field of study	IT	−0.520	15.384	0.000	2.272	−0.837	1.873	0.171	0.883
	Bus. Econ.	−0.125	0.388	0.533	0.594	0.821	5.887	0.015	0.433
Empl. status	Unemployed	−1.094	17.089	0.000	0.335	−0.368	5.334	0.021	0.692
Gender	Female	−0.833	14.332	0.000	0.435	−0.435	13.865	0.000	0.647
Age		−0.175	1.934	0.187	0.839	0.043	13.677	0.000	1.044
Constant		3.033	18.507	0.000	20.752	−0.433	1.349	0.245	0.649
Log-likelihood		−361.752				−944.522			
p		0.000				0.000			
Cox & Snell R Square		0,072				0072			
Nagelkerke R Square		0,168				0098			

Using binary logistic regression based on the p-value, we found that the variables Country (Kuwait), Level of study (Diploma), Employment status (Unemployed) and Age have a statistically significant impact on predicting the outcome of the dependent variable EQ1 (Table 2). Based on the Wald coefficient, we found that the variable Diploma has the greatest unambiguous impact on the prediction of the dependent variable EQ1. Considering that the odds ratio is less than 1, we conclude that it is more likely that the diploma student's answer will be incorrect.

The odds ratio in individual models presented in Table 2 (indicator C1.1) show that students in Kuwait are 53 % more likely to know the answer to question EQ1 than students in Serbia. For the modality Level of study, the data show that the probability of knowing the correct answer to EQ1 is 76 % lower for final year diploma students than for final year bachelor students, while the modality master students proved to be insignificant (p > 0.05). Employment status (40 % higher probability for unemployed students to know the answer) and Age (probability of students knowing the answer to EQ1 decreases by 3 % with each year) also proved to be statistically significant variables in the model (p < 0.05).

The socio-demographic characteristics of university students that determine the probability of students knowing the answer to question EQ2 are significantly different from EQ1. Table 2 shows that all variables except Age are statistically significant (p < 0.05). The Wald coefficient shows that the variable IT students has the greatest unambiguous impact on the prediction of the dependent variable EQ2 and that the answer of IT student is more likely to be incorrect since the odds ratio for this variable is less than 1.

The odds ratio indicate that students from Kuwait are 36 % less likely to know the answer to this question than students in Serbia, diploma students are 50 % less likely to know the answer than final year bachelor students, while master students are 114 % more likely to know the answer. In addition, students of IT and Business Economy are 68 % and 30 % less likely to answer the question correctly than Finance students. Unemployed students are 34 % less likely to answer the EQ2 question correctly than employed students, and female students are 37 % more likely than male students to answer the question correctly.

We can conclude that for the indicator C1.1.-Development of searches implementing information organisation and management mechanisms, no valid conclusion can be drawn about the relationship between different socio-demographic characteristics and the level of DEC due to the opposite direction and statistical significance in the individual models except for bachelor students in their final year of study. Final year bachelor students are likely to have higher levels of DEC for this indicator. This conclusion contrasts with the findings of Sookhtanio [82] for bachelor's and master's students in agricultural science and education, where master's students were found to have higher levels of information-seeking competences compared to bachelor's students. An important note is that this was found for different competence areas - agriculture and education.

Table 3 shows the model parameters for questions EQ3 and EQ4. Most of all variables in model EQ3 are statistically significant (p < 0.05), with the exception of Employment status (Unemployed), Age and Field of study (IT) (p > 0.05). As in the case of EQ1, the Wald coefficient shows that the variable Diploma has the greatest unambiguous impact on the prediction of the dependent variable EQ3, and since the odds ratio is < 1, we conclude that it is more likely that the diploma student's answer will be incorrect.

In model EQ4, the statistically significant variables (p < 0.05) are Country (Kuwait), Field of study (IT and Business Economy) and Gender (Female). The Wald coefficient indicates that the students of IT have the greatest unambiguous impact on predicting the answer to EQ4, which is more likely to be incorrect (odds ratio <1).

Based on the odds ratio, it can be concluded that students in Serbia are more likely to know the answer to EQ3, while Kuwaiti students are more likely to know the answer to EQ4. Bachelor's students are more likely than Diploma and Master's students to know the answer to EQ3, while the Level of study has no impact (p > 0.05) on the answer to EQ4. Business Economy students are more likely to know the correct answer to EQ3 than Finance students, who in turn are more likely to answer EQ4 than students in the other two fields of study. Female students are more likely to do better than male students on both EQ3 and EQ4.

For indicator C1.2.-Identification of entrepreneurship needs or opportunities within a virtual or based-on-technologies face-to-face environment - the conclusion is that female students are more likely to perform at a higher level. Existing evidence on the identification of entrepreneurial opportunities suggests the absence of differences in the innovativeness of identified opportunities between males and females [83] and that males discover more business opportunities than females [84]. Furthermore, gender does not play a role in opportunity-driven entrepreneurship [85].

From the test statistics presented in Table 5 and it can be seen that fewer of the variables included in the model EQ5 and model EQ6 are statistically significant compared to the other estimated models. The following variables are statistically significant (p < 0.05) in model EQ5: Country (Kuwait), Level of study (Master), Employment status (Unemployed) and Gender (Female), while the variables Country (Kuwait), Level of study (Diploma) and Gender are statistically significant in model EQ6 (p < 0.05). The Wald coefficient values indicate that variable Female in model EQ5 and variable Diploma in model EQ6 have the greatest unambiguous impact on the prediction of the dependent variables (EQ5 and EQ6) and the answers are more likely to be incorrect (odds ratio <1). The odds ratios for the statistically significant variables in both models indicate that male students from Serbia are more likely to know the correct answers to EQ5 and EQ6 respectively.

The low explanatory power of the estimated models for indicator 1.3. suggests that variables other than socio-demographic factors have a greater influence on the level of DEC under this indicator, which can be investigated in a separate study. We can conclude that male students from Serbia tend to have higher level of DEC for indicator 1.3.-Assessment of the limitations, opportunities and risks of potential entrepreneurship with technologies. Considering that the most important factor for the willingness to start a business is the ability to accept risks [86] and that females are considered more risk averse [87], this result for male students seems to be consistent with these arguments.

Odds ratio and p-value are suggesting that the acquired knowledge as a component of DEC (Indicator 2.1.) required to answer EQ7 and EQ8 is determined by Location (Serbia), Field of study (IT) and Level of study (Bachelor). Students are less likely to know the answer to EQ7 and EQ8 as they get older. For EQ8, employed students are more likely to know the correct answer. Based on the Wald coefficient, we found that in model EQ7 the variable Diploma and in model EQ8 the variable Kuwait have the greatest unambiguous impact on the answer prediction. Considering that in both cases the odds ratio is less than 1, we conclude that it is more likely that the answers will be incorrect.

The fact that for Indicator 2.1.-Specification of the most adequate digital contents and tools to respond to the possibilities found - IT and younger age students are more likely to have a higher DEC level seems rational given the nature of their studies, which are more technically oriented compared to Finance and Business Economy. This argument is in line with Margaryan, Littlejohn & Vojt [88] who confirmed that students of technical disciplines use more technological tools compared to other disciplines, and that business instructors should provide for greater and more effective use of technology in the class [89]. Furthermore, younger students tend to use technology more than their older peers, as noted by Staddon [90], which supports our findings on Indicator 2.1.

Although questions EQ9 and EQ10 are under the same EmDigital competence indicator (2.2.), the variables that are statistically significant in predicting their outcomes are completely different. The data in Table 6 show that the variables Gender and Age are not statistically significant for EQ9 (p > 0.05), while Level of study, Field of study and Age have no statistically significant effect on EQ10 (p > 0.05). The variables that have the greatest unambiguous impact on the answer prediction for EQ9 and EQ10 based on the Wald coefficient are IT students for EQ9 and Kuwait for EQ10 respectively. In both cases, the odds ratio (<1) indicates that the responses are more likely to be incorrect.

Due to the opposite directions (Country) and the statistical significance of the estimated variables, the only valid conclusion for Indicator 2.2 - Specification of ideas and opportunities in a creative manner is that employed students are more likely to have a higher DEC level. In the existing body of knowledge in this area, we have not found any research that addresses the relationship between specifying entrepreneurial ideas and opportunities in a creative manner and employment status that confirms or refutes our result. As mentioned earlier, Ferreras-Garcia et al. [46] have shown that variable experience has a positive impact on entrepreneurial competences in general, and this would be an argument that supports our finding.

The p-value for model EQ11 and model EQ12 targeting the sub-competence area Prospecting, indicate that the variables Country (Kuwait), Level of study (Diploma), Field of study (IT), Employment status (Unemployed) and Gender (Female) have a statistically significant impact on predicting the outcome of the dependent variable EQ11 and that the variables Level of study (Diploma and Master), Field of study (Business Economy), Employment status (Unemployed), Gender (Female) and Age have a statistically significant impact on predicting the outcome of the dependent variable EQ12 (Table 7). Using the Wald coefficient, we found that the variable Diploma for model EQ11 and variable Master for model EQ12 have the greatest unambiguous impact on predicting the response. Considering that for model EQ11 the odds ratio is less than 1, we conclude that it is more likely that the diploma student's answer will be incorrect, in contrast to model EQ12 where the odds ratio is above 1, indicating that the master student's answer is more likely to be correct.

For Indicator 3.1 - Exploration of the real possibilities of the development and implementation process of ideas within an immediate future team, the conclusion is that male students are more likely to have a higher level of DEC. The same applies for bachelor and employed students. The higher likelihood for male students to possess a higher level of DEC for this indicator is consistent with Ryu & Kim [91] who showed that business opportunity recognition is weaker in females than males. Employment status can again be related to experience [46] and the fact that the development of business opportunity prospection behavior is influenced by the type of organisation a person works for [92]. The reason that bachelor students are more likely to have a higher level of DEC for indicator 3.1. can be related to the longer educational cycle (4 years) compared to diploma students (2 years) and the positive effect of university education on this DEC competence indicator.

### 4.3 Association of the three sub-competence areas under identification of opportunities as the first competence area of EmDigital and different socio-demographic characteristics of university students

This research not only addresses the relationship between the socio-demographic characteristics of university students and Identification of opportunities as one of the four competence areas of DEC under EmDigital, but also seeks to explore the relationship between the three sub-competence areas under Identification of opportunities and various socio-demographic characteristics of university students. The previous sub-section explored the relationship between different DEC indicators under the first EmDigital competence area. In order to gain a more holistic insight and understanding of the relationship between different socio-demographic characteristics of university students and their DEC levels, the focus of this part of the analysis was on the sub-competence areas.

Table 8 shows the summary data of the effects of the variables and modalities studied on each indicator related to the different sub-competence areas 1) Search for and analysis of information, 2) Creativity and innovation, and 3) Prospecting. The positive sign in the table is associated with a higher probability of students knowing the correct answer related to the base modality and with higher DEC levels, while a negative sign is associated with a lower probability of students knowing the correct answer related to the base modality and consequently with a lower DEC levels. If no statistically significant difference was found between the observed and base modality of the variable under study, the fields are empty.

**Table 8:** Summarized results.

DEC competence	Question	Country	Level of studies		Field of studies		Employment status	Gender	Age
		Kuwait	Diploma	Master	IT	Business Economy	Unemployed	Female	
Search for and analysis of information	EQ1	+	–				+		–
	EQ2	–	–	+	–	–	–	+	
	EQ3	–	–	–		+		+	
	EQ4	+			–	–		+	
	EQ5	–		–			–	–	
	EQ6	–	–						
Creativity and innovation	EQ7	–	–	–	+				–
	EQ8	–	–	–	+	+	–	+	
	EQ9	+	+	+	–	–	–		
	EQ10	–					–	+	
Prospecting	EQ11	–	–		+		–	–	
	EQ12		–	–		–	–	–	+

Note: (−) is less likely for the student to know the answer to the question in relation to the base modality.

(+) is more likely for the student to know the answer in relation to the base modality.

The results show that for the first sub-competence area, Search for and analysis of information, the country in which the student attends university has a statistically significant impact on the competence level. Students from Serbia are more likely to perform better in this sub-competence area than students in Kuwait (students in Kuwait are less likely to know the correct answers in 4/6 cases than their peers in Serbia). Diploma students are also more likely to perform worse in this sub-competence area (lower probability in 4/4 cases) than Bachelor students, and the same statement can be made for Master students (2/3 cases with lower probability compared to Bachelor students). The results also indicate that Finance students will do better than Business Economy (lower probability for Business Economy students in 2/3 cases) and IT students (lower probability for IT students in 2/2 cases). The variable Gender also has a statistically significant impact on the competence level in this sub-competence area - female students are more likely to perform better than male students (higher probability for female students in 3/4 cases). In summary, in this sub-competence area, female and finance and bachelor students are more likely to have a higher level of DEC in terms of searching for and analysing information for their digital ventures. Brixiova, Kangoye & Said [93] provide evidence that tertiary education increases the impact of financial literacy training on women's entrepreneurial performance, which is consistent with our findings. Location also plays an important role in this sub-competence area - students from Serbia are more likely to have higher levels of DEC.

For the second sub-competency area, Creativity and innovation, the variables Country, Level of study and Gender have almost the same impact on the competence as in the case of the first sub-competence area. The identified effects are slightly different for the variables Field of study and Employment status. IT students are more likely to perform better (higher probability for IT students in 2/3 of the cases) than Finance students and no valid conclusion can be drawn for Business Economy students (1 case with lower probability and 1 case with higher probability). Employed students are more likely to have a higher level of competence in this sub-competence area than unemployed students (lower probability for unemployed students in 3/3 cases). In summary, the level of DEC in the sub-competence area Creativity and innovation is mainly influenced by location (Serbia) and employment status (Employed), followed by gender (Female), field of study (IT) and level of study (Bachelor). Higher levels of entrepreneurial creativity among employed students are a consequence of the fact that “students who juggle working and studying develop greater skills and abilities, have a different outlook on things, a greater ability to resolve problems or propose different solutions” [94]. The fact that female students are more likely to have higher levels of competences related to creativity and innovation is in line with the research of Ferreras et al. [95], which proved that female students achieve better innovation competences than their male peers and in contrast to the research of AlShobaki et al. [96]. The latter showed that there are no differences in entrepreneurial creativity between male and female students.

Considering that the sub-competence area Prospecting only has one indicator (3.1.), all the results and conclusions presented in the previous sub-section for indicator 3.1. also apply to the sub-competence area Prospecting. The probability of having the highest level of competence in this sub-competence area applies to employed, male, and bachelor students. Unemployed, female and diploma students had a lower probability of knowing the correct answer in 2/2 cases.

## 5 Conclusions

The research findings in this article target two aspects of DEC of university students - DEC levels and the association of DEC levels with various socio-demographic characteristics of university students, in line with identified research questions in Section 1.

Based on the results representing the DEC levels of the first competence area of EmDigital (Identification of opportunities) and the three sub-competence areas under it (Search for and analysis of information, Creativity and innovation, and Prospecting), the general conclusion can be drawn that the DEC levels of the students participating in this research are low overall. The mean value of the DEC level of students for the whole sample is 34.5 %, which suggests that more attention and a different approach to contemporary education on digital entrepreneurship are needed, at least in the countries that participated in this research. Students in Serbia performed better compared to students in Kuwait. The level of acquired DEC (identification of opportunities) is higher for Bachelor students than for Diploma and Master students. In terms of field of study, Finance students showed better knowledge than IT and Business Economy students. Employed students performed better than unemployed students, and female students performed slightly better than male students.

The results showing the relationship between the socio-demographic characteristics of university students and the levels of their DEC were presented separately for the entire competence area Identification of opportunities and the three sub-competence areas in it - Search for and analysis of information, Creativity and innovation, and Prospecting.

The results proved the relationship between different socio-demographic characteristics of university students and DEC competence levels. For the overall competence area Identification of opportunities, as the first competence area of EmDigital, the positive association with the following socio-demographic variables of university students was confirmed: Location (Serbia), Level of study (Bachelor), Field of study (IT and Finance), Employment status (employed) and Gender (female). The students with the above characteristics are more likely to have a higher level of DEC for this competence area.

The results for each sub-competence area show a slightly different relationship between the different socio-demographic characteristics of the students, which is understandable given that each sub-competence focuses on a separate area - Search for and analysis of information, Creativity and innovation, and Prospecting. The level of students DEC in Search for and analysis of information as the first sub-competence area under Identification of opportunities are associated with Location, Level of study, Field of study and Gender. A bachelor in Finance and female students are more likely to have higher DEC levels for this sub-competence. For the second sub-competence area, Creativity and innovation, Location (Serbia) and Employment status (employed) were found to have the most impact. The third sub-competence area Prospecting, is associated with Level of study (bachelor), Employment status (employed) and Gender (male).

The research presented in this paper makes several theoretical contributions to the existing literature on DEC in general and for university students DEC in particular. This research addresses the lack of research on the level of DEC for any target population and fills the gap in the academic literature identified by Garcez et al. [41] who proposed a future study aimed at measuring the level of DEC at the time students leave the university programme. In addition, this work represents the first application of the DEC competence model (framework) EmDigital developed by Prendes-Espinosa et al. [21], and in particular the first competence area identification of opportunities. The results presented in this paper represent the first empirical evidence on the level of university students DEC for the specified competence area, using the EmDigital framework. In line with findings on the relationship between DEC and DEI [26,27] and studies that assessed EC of university students [35] and examined the relationship between levels of EC and various socio-demographic characteristics of university students [36] to examine the success of university-level entrepreneurship education in creating EC as a critical determinant in building students' EI, this study contributes to the assessment of DEC and associating their levels with various socio-demographic characteristics of university students (as suggested by Alferaih [26]) to investigate the success of university-level digital entrepreneurship education in creating DEC. Moreover, this study responds to a call for future research aimed at creating entrepreneurial competences in the Digital “University based” Entrepreneurial ecosystems proposed by Secundo et al. [40]. An important step in the creation of DEC is its assessment as a feedback mechanism for the success of the Digital “University based” Entrepreneurial ecosystems in creating DEC.

The practical implication of this research lies in the fact that it has provided the first empirical evidence of the level of DEC among university students. The findings could serve the universities better understand the impact of their digital entrepreneurship education efforts and take corrective action in their curricula to better align curricula, teaching methods and student learning outcomes with the demands of today's labour market. Understanding the relationship between the different sub-competence areas under the Identification of opportunities and the socio-demographic characteristics of university students will also allow universities to use a more personalised approach to teaching digital entrepreneurship according to students' characteristics and inclinations towards different DEC.

The limitations of this study can be summarized as follows. Although the questionnaire used in this study was pre-tested, there is a possibility that not all respondents in a sample of students in Kuwait and Serbia understood the questions in the same manner. The second limitation is related to the fact that the indicators used in the EmDigital model cannot fully measure (with 100 % accuracy) the DEC acquired. Finally, the chosen econometric specification of the model used does not describe the relationship between the dependent and the selected explanatory variables with 100 % accuracy, as in any other econometric analysis.

The research presented in this paper focused on EmDigital's first competence area - Identification of Opportunities. Future research should examine all four competence areas of EmDigital and provide a full insight into the DEC levels and how they relate to the socio-demographic characteristics of university students. Furthermore, in addition to the explanatory variables included in the model, other independent variables that are likely to have higher explanatory power should be investigated and included in the model, and/or other socio-economic and demographic variables included in the model could increase the value of pseudo R2.

## Data availability statement

Data will be made available on request.

## Ethics statement

This study was reviewed and approved by Scientific Research Centre of the Australian University in Kuwait with the approval number: SRC01-05/2021.

All participants provided informed consent to participate in the study.

## CRediT authorship contribution statement

**Vladimir Simovic:** Conceptualization, Data curation, Formal analysis, Funding acquisition, Investigation, Methodology, Supervision, Writing – original draft, Writing – review & editing. **Ivana Domazet:** Conceptualization, Formal analysis, Investigation, Methodology, Project administration, Resources, Writing – original draft, Writing – review & editing. **Milica Bugarcic:** Formal analysis, Investigation, Methodology, Writing – original draft, Writing – review & editing, Data curation. **Mirna Safi:** Investigation, Resources, Supervision, Writing – original draft, Writing – review & editing. **Hamsa Sarhan:** Investigation, Resources, Writing – original draft, Writing – review & editing. **Rupali Bhagat:** Investigation, Resources, Writing – original draft, Writing – review & editing. **Aleksandra Bradic Martinovic:** Conceptualization, Methodology, Writing – review & editing.

## Declaration of AI and AI-assisted technologies in the writing process

During the preparation of this work the authors used InstaText in order to improve language and readability of the paper. After using this tool/service, the authors reviewed and edited the content as needed and take full responsibility for the content of the publication.

## Declaration of competing interest

The authors declare the following financial interests/personal relationships which may be considered as potential competing interests:Vladimir Simovic reports financial support, article publishing charges, and equipment, drugs, or supplies were provided by Kuwait Foundation for the Advancements of Sciences (10.13039/501100003286KFAS).

## Figures and Tables

**Table 1:** Identification of Opportunities as the first competence area of EmDigital

Competence Area	Sub-Competence	Indicator
Identification of Opportunities	C1. Search for and analysis of information	C1.1. Development of searches implementing information organization and management mechanisms.
		C1.2. Identification of entrepreneurship needs or opportunities within a virtual or based-on-technologies face-to-face environment.
		C1.3. Assessment of limitations, opportunities and risks of potential entrepreneurship with technologies.
	C2.Creativity and innovation	C2.1. Specification of the most adequate digital contents and tools to respond to the possibilities found
		C2.2. Specification of ideas and opportunities in a creative manner
	C3. Prospecting	C3.1. Exploration of the real possibilities of the development and implementation process of ideas within an immediate future team.

**Table 2:** Legend

Code	Label
EQ1	You are trying to identify the targeted audience(s) in a B2C segment for your digital business idea. In order to do it, you use.
EQ2	Where can you find useful information about your competitors using online search?
EQ3	A digital business model in which a part of features is free to use by the customer and the more advanced features need to be paid is called …
EQ4	For the purpose of testing the business idea and setting a value proposition of the digital business you will use …
EQ5	Distributed Denial of Service is a type of criminal activity associated with …
EQ6	Name an emerging technology and briefly explain how it can be used as a basis for digital business
EQ7	Research results show that product brochures with prices need to be on the website to earn more profit while gaining new customers. To make an eye-catching brochure you would use …
EQ8	You are looking to inform via email all your loyal customers about the pre-sale of some products/services of your digital business. Which tool would you use?
EQ9	Revenue streams, Cost structure and Key Activities are a part of the tool which is being used for the purpose of specifying digital business ideas. This tool is …
EQ10	If you were asked to specify the MVP (Minimum Viable Product) for your digital product or service, you would do one of the following …
EQ11	You should specify the budget for launching your new digital product or service. Explain how you would do so.
EQ12	You should identify the profitability of your customers in a customer portfolio of your company. Which technique would you use?
